# An Insight by Molecular Sensory Science Approaches to Contributions and Variations of the Key Odorants in Shiitake Mushrooms

**DOI:** 10.3390/foods10030622

**Published:** 2021-03-15

**Authors:** Si-Lu Wang, Song-Yi Lin, Han-Ting Du, Lei Qin, Li-Ming Lei, Dong Chen

**Affiliations:** School of Food Science and Technology, National Engineering Research Center of Seafood, Collaborative, Innovation Center of Seafood Deep Processing, Dalian Polytechnic University, Dalian 116034, China; wangsl2021@163.com (S.-L.W.); linsongyi730@163.com (S.-Y.L.); duhanting960415@163.com (H.-T.D.); qinlei@dlpu.edu.cn (L.Q.); llm3194@163.com (L.-M.L.)

**Keywords:** odorants, molecular sensory science, aroma recombination verification, solvent-assisted flavor evaporation (DSE-SAFE), headspace solid phase microextraction (HS-SPME), aroma extract dilution analysis (AEDA), detection frequency analysis (DFA), odor activity value (OAV), gas chromatography-olfactometry (GC-O), gas chromatography-mass spectrometry (GC-MS)

## Abstract

An insight using molecular sensory science approaches to the contributions and variations of the key odorants in shiitake mushrooms is revealed in this study. Odorants were extracted by headspace solid phase microextraction (HS-SPME) and direct solvent extraction combined with solvent-assisted flavor evaporation (DSE-SAFE) in fresh and hot-air-dried shiitake mushrooms. Among them, 18 and 22 predominant odorants were determined by detection frequency analysis (DFA) and aroma extract dilution analysis (AEDA) combined with gas chromatography-olfactometry (GC-O) in the fresh and dried samples, respectively. The contributions of these predominant odorants in the food matrix were determined by quantification and odor activity values (OAVs) with aroma recombination verification. There were 13 and 14 odorants identified as key contributing odorants to overall aroma, respectively. 1-Octen-3-ol and 1-octen-3-one were the most key contributing odorants in the fresh samples in contributing mushroom-like odor. After hot-air-drying, the OAV and concentrations on dry basis of the key contributing odorants changed, due to oxidation, degradation, caramelization and Maillard reactions of fatty acids, polysaccharides and amino acids. 1-Octen-3-ol was reduced most significantly and degraded to 1-hydroxy-3-octanone, while phenylethyl alcohol increased the most and was formed by phenylalanine. In hot-air-dried samples, lenthionine became the most important contributor and samples were characterized by a sulfury odor. Overall contributions and variations of odorants to the aroma of shiitake mushrooms were revealed at the molecular level.

## 1. Introduction

Shiitake mushrooms (*Lentinus edodes*), belonging to fungi phylum tricholoma, are one of most important traditional delicacies and medicinal fungi in Asia on account of their nutritional characteristics, medicinal properties and distinctive flavor [1,2]. They hold the second highest market share in the global market consumption of edible fungi [3]. Generally, shiitake mushrooms are rich in proteins, vitamins, minerals, and low in fat and cholesterol [3,4]. In addition, they have the medicinal properties of being anti-tumoral, anti-microbial, lowering cholesterol activity, lowering blood pressure, improving liver function and strengthening the immune system [5,6]. What is more, shiitake mushrooms are mainly popular for their unique flavor properties and are used as a basis for various dishes [7,8]. 

Due to their short shelf-life, shiitake mushrooms are usually dehydrated for preservation. Hot-air-drying is the most widely used method for production of dehydrated fruits and vegetables, not only because it is low cost, with easy control and short process time, but also because it contributes a unique flavor [9,10,11]. In recent years, researchers have studied the changes induced by hot-air-drying in the volatile composition of shiitake mushrooms. The total content of volatiles increased significantly after hot-air-drying [12]. Hot-air-drying caused the loss of C8 compounds and straight chain sulfur compounds and increased cyclic sulfur compounds [13]. Qin et al. [14] studied the aroma profile of shiitake mushrooms during hot-air-drying. However, their study only depended on a volatile and sensory analysis, and the odorants and their contributions to the aroma were not reported. Therefore, odorants need to be qualified and quantified by an advanced flavor analysis method to reveal the contributions and variations of odorants in shiitake mushrooms at the Sensomics molecular level.

Molecular sensory science, which is also known as Sensomics, is a systematic science with which to study food odorants qualitatively and quantitatively at the molecular level [15]. This systematic approach consists of separation, extraction, identifying the aroma property, quantifying odor contribution and verification.

The common techniques applied to extract or isolate odorants in recent years are steam distillation/extraction (SDE), headspace solid phase microextraction (HS-SPME), and direct solvent extraction combined with solvent-assisted flavor evaporation (DSE-SAFE). SDE is considered to be a relatively convenient and simple method with high extraction capacity and recovery [16]. It has been widely used in previous aroma study, such as on *Maclura tricuspidata* fruit, onion oil and porcini [17,18,19]. However, the authenticity of the odorants is affected by the elevated temperatures during SDE [20]. HS-SPME is a sensitive, fast and solvent-free technique with strong enrichment ability and suitable for highly volatile compounds [16,21]. DSE-SAFE is a low-temperature and mild isolation process for volatile and semi-volatile compounds, which can minimize the alterations of volatiles during extraction and remove the non-volatile compounds in solvent prior to analysis by gas chromatography-mass spectrometry (GC-MS) [21,22]. Therefore, SPME and DSE-SAFE will be combined in this study to isolate and extract odorants exactly and thoroughly.

The aroma property and strength of each odorant can be identified by gas chromatography-olfactometry (GC-O), which is based on the use of human assessors as sensitive and selective detectors to ascertain odorants in a sample extract [23]. Detection frequency analysis (DFA) and aroma extract dilution analysis (AEDA) are the methods used to identify the major odorants combined with GC-O, and are widely used in rice, fruit, wine and other food substrates [24,25,26]. DFA is simple, repeatable and time-saving, and panels may not even have to be trained prior to the analysis [27]. AEDA is suitable for screening potent odorants, and can be used to determine the sensory importance of medium and high boiling point odors in solvent extracts [28,29]. Based on the predominant odorants identified by DFA and AEDA, odor activity values (OAVs) can reasonably estimate their contributions to overall aroma characteristics in food matrix, which is determined by the threshold and the concentration of the odorant [30,31]. 

The objective of the present study was to (I) identify the odorants extracted by HS-SPME and DSE-SAFE and determine the predominant odorants by application of DFA and AEDA in fresh and hot-air-dried shiitake mushrooms; (II) determine the contributions of the predominant odorants to the aroma of fresh and hot-air-dried shiitake mushrooms by means of internal and external standard quantification and OAVs with aroma recombination model; (III) characterize the changes of the key contributing odorants induced by hot-air-drying and the aroma chemistry. These results might provide an insight to the contribution of odorants to mushroom flavor and present a new contribution to food flavor study.

## 2. Materials and Methods

### 2.1. Materials

Fresh shiitake mushrooms (L-808, cultivated in northeast China) were purchased from a local market in Dalian, Liaoning province, China. Shiitake mushrooms with similar size and without mechanical damage were picked out for analysis. The pilei of shiitake mushrooms were further divided into four equal parts.

### 2.2. Chemicals

Diethyl ether (anhydrous, 99.7%), sodium chloride (NaCl) and sodium sulfate (granular, anhydrous 99%) were purchased from Sangon Biotech (Shanghai) Co., Ltd. (Shanghai, China). n-Alkane standards (C7-C30) and internal standards: 2-ethylbutanoic acid (acid fraction), 2-methyl-3-heptanone (neutral fraction), 2-nonanol (alcohol fraction) and 2,4,6-trimethylpyridine (basic fraction) were purchased from Sigma-Aldrich Chemicals Co. (St. Louis, MO, USA). Authentic reference compounds listed in (Tables 1–3) were obtained from commercial sources, as follows: nos. 1–4, 8, 10, 12, 14–18, 20–25, 30, 33 (Aladdin, Shanghai, China); no. 5 and 11 (TCI, Shanghai, China); nos. 6, 7, 19, 27, 32, 34, 35, 37, 41 (Sigma-Aldrich, Shanghai, China).

### 2.3. Hot-Air-Drying Processing

The drying process was carried in a constant-temperature drying oven (DHG-9053A, Jinghong Laboratory Equipment Co., Shanghai, China). The samples were hot air dried at 60 °C in the oven with an air flow speed of 1 m/s and an air humidity of 10%. The samples were spread in a single layer on the tray. The drying process lasted for 10 h until the samples reached constant weight. The water content determination was replicated three times and the average was reported. The water content of pilei was 3.94 ± 0.33% (on wet basis) at 10 h, meeting the national food safety standards for edible fungi and its products (GB7096-2014). After the drying was completed, the dried products were kept in sealed aluminum foil bags and stored at −80 ℃ until analysis. 

### 2.4. HS-SPME Analysis

Chopped samples (0.5 g) with saturated NaCl (4 mL) solution were hermetically sealed in a 20 mL headspace glass having a PTFE/silicone septum (Supelco, Bellefonte, PA, USA) and a magnetic screw cap. Then the samples were incubated at 40 °C for 40 min. A stainless steel needle containing divinylbenzene/carboxen/polydimethylsiloxane (DVB/CAR/PDMS, 50/30 μm) SPME fiber (Supelco, Bellefonte, PA, USA) was inserted through the septum of the sample vial to extract the volatile compounds at 40 °C for 40 min. The sample vials were shaken in the agitator at 250 rpm/min during incubating and extracting.

### 2.5. DSE-SAFE Analysis

The samples were extracted following Erten and Cadwallader [21] with some modifications. The fresh (150 g) and dried (18.05 g) shiitake mushrooms with equal contents on a dry basis were broken, and extracted in a PTFE centrifuge bottle with diethyl ether. Then the ether layer was collected by centrifugation. Extraction and centrifugation were repeated twice more with fresh diethyl ether. Subsequently, the extracts were subjected to solvent assisted flavor evaporation (SAFE), and concentrated using the Vigreux column (45 ℃).

### 2.6. Gas Chromatography-Olfactometry (GC-O)

GC-O analysis was performed using an Agilent 7890B GC (Agilent Technologies Inc., Santa Clara, CA, USA) equipped with a flame ionization detector (FID), a sniffing port (ODP3; Gerstel, Mülheim an der Ruhr, Germany), a cool on-column injector and a split/splitless (S/SL) injector. Separations were performed on a fused silica capillary column (either Rtx®-Wax, 30 m length × 0.32 mm i.d. × 0.25 µm film, Restek; or HP-5, 30 m length × 0.32 mm i.d. × 0.25 µm film; Agilent Technologies Inc., USA). The sample (1.0 µL) extracted by DSE-SAFE was injected cold on-column at 35 °C in track oven mode, and that extracted by HS-SPME was injected S/SL injector at 250 °C in splitless mode. Nitrogen was used as carrier gas at a fixed flow rate of 1.53 mL/min. The oven temperature was programmed from 35 °C, increased by 2 °C/min to 130 °C, then increased to 250 °C at 8 °C/min and held for 10 min. The flow of the carrier gas was split in a 1:1 ratio at the end of the capillary column. One part was directed to FID (250 °C) and the other to the sniffing port (200 °C). A series of n-alkanes C7-C30 for the DB-wax and the HP-5 was used to determine linear retention indices (RI).

### 2.7. Detection Frequency Analysis (DFA)

DFA using a panel of four judges (two males and two females) was applied to obtain the odor pattern of shiitake mushrooms following the methodology described by Pang et al. [32]. In total, eight GC-O runs were performed (two runs for each assessor). The detection frequency (DF) for an odor with the same RI and a similar description was summed. At the sniffing port any odorant that had total DF ≥ 2 (reported by at least two assessors) was arbitrarily considered to have aroma potential activity.

### 2.8. Aroma Extract Dilution Analysis (AEDA)

AEDA was used to detect the potential odorants of the DSE-SAFE isolates. The concentrated extract was diluted stepwise in a series of 1:3n with diethyl ether. Each dilution was performed using the GC-O conditions described above until no odor was detected. Flavor dilution (FD) factor of the odorants at the sniffing port was used as a measure for the intensity of a compound, and the higher FD factors were concluded to have a greater relative importance [33].

### 2.9. Gas Chromatography-Mass Spectrometry (GC-MS)

The odorants were determined by the Agilent 7890B/5977B GC-MS instrument (Agilent Technologies Inc., USA) with VF-WAXms and HP-5MS capillary columns (both 30 m × 0.25 mm × 0.25 μm, Agilent Technologies Inc., USA). The sample (0.4 μL) extracted by DSE-SAFE was injected in split ratio (10:1), and that extracted by HS-SPME was injected in splitless mode, with the inlet at 250 °C. The carrier gas was 99.999% pure helium at a fixed flow rate of 1.68 mL/min. The initial oven temperature was 35 °C, ramped at 2 °C/min to 130 °C, then increased to 250 °C at 8 °C/min and held for 10min. Mass detector was conducted in an electron impact mode at 70 eV and the ion source temperature was 230 °C. Mass spectra (MS) were scanned from 50 to 550 amu. 

### 2.10. Identification of Odorants

Odorants were positively identified by matching several criteria of unknown odorants to authentic reference standards, including odor characteristics, retention index (RI, on polar and nonpolar GC columns) and electron-impact mass spectra (MS). If one or more of the above criteria could not be met, the compound was considered tentatively identified. RI was calculated by injection of series of n-alkanes as described by Van Den Dool and Kratz [34].

### 2.11. Quantitation of the Predominant Odorants

Quantitation was performed following Zhang et al. [20] with some modifications. Positively identified odorants exhibiting high FD factors (FD ≥ 3) or DF factors (DF = 8) were quantitated by constructing standard curves, which were the linear regressions of mass ratio (target/internal standard) of standard substance versus selected ion area ratio (target/internal standard) [21]. Odorants extraction was performed as above described except that shiitake mushroom samples were first spiked with 800 μL mixture of internal standards (0.01 mg/mL). Mixed internal standards with five levels of concentration were prepared for the calibration and standards were analyzed in triplicate. These solutions were detected by GC-MS as described in Section 2.9, except that mass spectrometry was conducted in the single ion monitoring (SIM) mode. The concentration of a target compound was determined based on the ratio of its selected ion peak area to that of selected ion peak area of its corresponding internal standard (IS) (Table 3), and the formula as follows:

(1) $$\mathrm{concentration}\;(\mu g/g,\;d.b{.)\;=\;(\mathrm{area}}_{\mathrm{target}}{/\mathrm{area}}_{\mathrm{IS}})\;\times \;\mathrm{Rf}\;\times \;\mathrm{mass}\;\mathrm{of}\;\mathrm{IS}\;(\mu g\;)/\mathrm{sample}\;\mathrm{mass}\;(g,\;d.b.)$$

Rf was the response factor (1/slope).

### 2.12. Calculation of Odor Activity Values (OAV)

The OAV of an odorant was determined by dividing its concentration by its published odor detection threshold in water [21].

### 2.13. Aroma Analysis

Aroma was determined by 20 experienced panelists. The judgements of the panelists were averaged. Broken fresh shiitake mushrooms (20 g) and dry shiitake mushrooms powder (2 g) were presented in covered and odorless plastic vessels at room temperature. The assessors were asked to evaluate the intensities of selected characteristic aromas (mushroom, grass, metallic, sulfury, caramel, fatty, and cabbage for fresh shiitake mushrooms; mushroom, chocolate, sulfury, caramel, sweaty, seasoning-like, and cooked potato-like for dry shiitake mushrooms). The intensities were ranged on a six-point scale from 0 (not perceivable) over 1, 2, ..., to 5 (strongly perceivable). The descriptors were compared with aqueous solutions of the reference odorants, and the concentrations of the reference odorants in different odor intensities are shown in (Table 1).

### 2.14. Aroma Recombination Experiments.

All odorants with OAVs ≥ 1 were prepared by mixing them in water solution at their actual concentrations determined in the fresh and hot-air-dried shiitake mushrooms. Then the solution was added to the deodorized matrix, which was prepared using the method of Zhang et al. [20]. Subsequently, the recombination samples, the fresh and hot-air-dried shiitake mushrooms, were each placed in closed, odorless plastic vessels and evaluated by the panelists as explained in Section 2.13. 

### 2.15. Statistical Analysis

All statistical analyses were performed in triplicate, with the experimental results expressed as means ± SD. The data were analyzed using the Statistical Program for Social Sciences (SPSS 20.0, Chicago, IL, USA) software for analysis of variance and Duncan’s test. The significance was established at *p* < 0.05. The aroma recombination experiments data were collected and analyzed by Excel (Microsoft Office 2018, Redmond, WA, USA).

## 3. Results and Discussion

### 3.1. Odorants in Fresh Shiitake Mushrooms

#### 3.1.1. Odorants Identified by HS-SPME

A total of 23 odorants were detected by HS-SPME in the fresh shiitake mushrooms (Table 2). Among them, 17 compounds were positively identified, six compounds were tentatively identified and three compounds were unknown. Most odorants were related to mushroom, herbaceous tinge and sulfury descriptors. 

Thirteen compounds were detected by DFA in the fresh samples by all assessors (DF = 8), indicating that they contributed more actively to the aroma of fresh samples and were considered as the predominant odorants. These consisted of four aldehydes, four sulfur compounds, two alcohols, two ketones, and one nitrogen-containing compound. Aldehydes, sulfur compounds, alcohols and ketones have been reported as the main volatile compounds in fresh shiitake mushrooms [35,36]. In addition, 3-octanone, 1-octen-3-one and 1-octen-3-ol exhibited a mushroom-like odor, which was the main odor attribute of shiitake mushrooms. The sulfur compounds, such as 3-(methyl-thio)-1-propanal, 3-(methyl-thio)-1-propanol, 1,2,4,5-tetrathiane and lenthionine, were also found as predominant odorants in fresh samples and offered cooked potato-like, cabbage-like, sulfury and burnt aroma properties. 

#### 3.1.2. Odorants Identified by DSE-SAFE

As shown in (Table 2), 21 odorants were positively and seven compounds tentatively identified in fresh shiitake mushrooms by DSE-SAFE. Three compounds were not identified. 

Twenty-three odorants exhibited high FD factors (FD ≥3) by AEDA, indicating that these 23 compounds made major contributions to the overall aroma and were the predominant odorants of fresh shiitake mushrooms. Among these, phenylacetaldehyde (floral), *trans*-4,5-epoxy-(E)-2-decenal (metallic), 1,2,4,5-tetrathiane (sulfury, burnt), 1,2,4,6-tetrathiepane (sulfury) and lenthionine (sulfury, burnt) had the highest FD factors of 729, followed by 3-methyl-butanal (chocolate), 3-(methyl-thio)-1-propanal (cooked potato-like), dimethyl tetra-sulfide (mushroom) with FD factors of 243. The relatively high FD factors of sulfur compounds, like 1,2,4,5-tetrathiane, 1,2,4,6-tetrathiepane, lenthionine, 3-(methyl-thio)-1-propanal and dimethyl tetra-sulfide, could demonstrate that the sulfur compounds also provided major aroma properties to fresh samples. However, Tian et al. [12] reported that dimethyl tetra-sulfide and 1,2,4,6-tetrathiepane were not detected in fresh shiitake mushrooms. This might be due to the different types of GC column and detector. Only the polar column was used in the study of Tian et al. [12], while dimethyl tetra-sulfide and 1,2,4,6-tetrathiepane were identified as the predominant odorants on the nonpolar column in this study. Therefore, the use of columns with different polarities could more comprehensively identify odorants, since there were many types of aroma compounds with different polarities in the food matrix. In addition, 1-octen-3-one and 1-octen-3-ol were also more important odorants in fresh samples with FD factors of 81, which was consistent with the result identified by DFA. Furthermore, only 13 compounds were consistent with the odor-active compounds detected by Schmidberger and Schieberle [3] in raw shiitake mushrooms. This difference might be due to the different varieties, cultural practices and collection time of shiitake mushrooms, and the distinct extraction and identification methods applied [37].

### 3.2. Odorants in Dried Shiitake Mushrooms

#### 3.2.1. Odorants Identified by HS-SPME

Among the 25 odorants detected by HS-SPME in the dried shiitake mushrooms, 19 compounds were positively identified, six compounds were tentatively identified, and only one compound was not identified (Table 2). Most odorants were related to mushroom-like, sulfury, sweet and sweaty descriptors. Based on DFA, 15 compounds had DF of 8, illustrating that these components were detected in the dried shiitake mushrooms by all assessors. Although Tian et al. [12] reported that sulfur compounds and acids were the main volatile compounds in dried shiitake mushrooms, the sulfur compounds and acids were further demonstrated in this study as the predominant odorants in the dried samples, and not only the main volatiles. The sulfur compounds, including dimethyl trisulfide, 1,2,4,5-tetrathiane and lenthionine, performed higher DF factors and contributed to a stronger sulfury odor to dried samples. The acids, like isovaleric acid and phenylacetic acid, contributed sweaty and floral odors, respectively. 

#### 3.2.2. Odorants Identified by DSE-SAFE

In the dried shiitake mushrooms, 31 odorants were detected by DSE-SAFE, of which 24 were positively identified, seven were tentatively identified, and two were unknown. Among these 31 odorants, 25 odorants showed their relatively high FD factors (FD ≥ 3) (Table 2). And dimethyl tetra-sulfide (mushroom), 4-hydroxy-2,5-dimethyl-3(2H)-furanone (caramel), 1,2,4,5-tetrathiane (sulfury, burnt) and lenthionine (sulfury, burnt) showed the highest FD factors of 729, followed by 3-methyl-butanal (chocolate) with FD factor of 243. Additionally, 2-methyl-propanal (chocolate), 1-hexanol (sweet, oily), 2-ethyl-1-hexanol (fresh, fatty), (E)-2-octen-1-ol, phenylacetaldehyde (floral), 3-(methyl-thio)-1-propanol (cabbage), phenylethyl alcohol (honey) and benzoic acid (balsam-like) were identified for the first time as predominant odorants in the dried shiitake mushrooms.

### 3.3. Concentrations and Odor-Activity Values (OAVs) of Positively Identified Predominant Odorants in Shiitake Mushrooms

Concentrations and OAVs of 26 positively identified predominant odorants determined in fresh and dried shitake mushrooms are given in (Table 3). Among the 18 predominant odorants quantitated in the fresh shiitake mushrooms, 1-octen-3-ol was the most abundant with 157.65 ± 1.17 µg/g, which accounted for approximately 94% of total odorants, followed by (E)-2-octenal (1.70 ± 0.09 µg/g), 3-methyl-butanal (1.39 ± 0.07 µg/g) and 3-octanone (1.34 ± 0.03 µg/g). Murray et al. [38] reported that 1-octen-3-ol had the highest concentration and (E)-2-octenal also had higher content in the American matsutake. 1-Octen-3-ol had the highest OAV of 105100, followed by 1-octen-3-one (36547) and lenthionine (4048), which made major contributions to the aroma of fresh shiitake mushrooms. 1-Octen-3-ol, 3-methyl-butanal and lenthionine had higher concentrations, as well as higher OAVs in the fresh samples. However, 1-octen-3-one and *trans*-4,5-epoxy-(E)-2-decenal had lower contents, but relatively higher OAVs. This might be due to the fact that there was a strong correlation between odor thresholds of aroma compounds and their aroma contribution except concentrations, and the OAV was defined as the ratio of the aroma concentration to its odor threshold [31,39]. 

**Table 3:** Quantification of the predominant odorants in fresh and hot-air-dried shiitake mushrooms.

No.	Compound	Internal Standard	Target Ion ^a^	IS Ion ^b^	Response Factor	*R* ^2 c^	Concentrations (µg/g)		Odor Threshold in Water (µg/g)	OAV ^d^	
							Fresh	Dried		Fresh	Dried
Esters											
2	Ethyl acetate	2-Methyl-3-heptanone	61	57	5.8754	0.9940	-	218.34 ± 16.35	5^e^	-	44
Aldehydes											
3	3-Methyl-butanal	2-Methyl-3-heptanone	58	57	8.6580	0.9999	1.39 ± 0.07	0.43 ± 0.04	0.0011 ^f^	1263	394
7	Octanal	2-Methyl-3-heptanone	84	57	8.6356	0.9995	0.45 ± 0.06	-	0.0034 ^g^	134	-
12	(E)-2-Octenal	2-Methyl-3-heptanone	55	57	6.2578	0.9950	1.70 ± 0.09	-	0.003 ^e^	568	-
18	Benzaldehyde	2-Methyl-3-heptanone	106	57	0.7579	0.9998	0.02 ± 0	0.83 ± 0.04	3.5 ^h^	<1	<1
23	Phenylacetaldehyde	2-Methyl-3-heptanone	91	57	0.7399	0.9982	0.34 ± 0.02	1.69 ± 0.13	0.004 ^e^	86	422
32	*trans*-4,5-Epoxy-(E)-2-decenal	2-Methyl-3-heptanone	81	57	29.1545	0.9984	0.13 ± 0.02	-	0.00013 ^g^	1033	-
Ketones											
4	2,3-Butanedione	2-Ethylbutyric acid	86	88	1.0495	0.9947	-	0.04 ± 0	0.001 ^g^	-	43
6	3-Octanone	2-Methyl-3-heptanone	99	57	1.5314	1	1.34 ± 0.03	0.01 ± 0	0.0214 ^f^	63	<1
8	1-Octen-3-one	2-Methyl-3-heptanone	55	57	1.2860	0.9997	0.58 ± 0.02	0.03 ± 0	0.000016 ^g^	36,547	1989
Sulfur compounds											
5	Dimethyl disulfide	2-Methyl-3-heptanone	94	57	0.8281	0.9967	-	1.42 ± 1.11	12 ^i^	-	<1
11	Dimethyl trisulfide	2-Methyl-3-heptanone	126	57	0.6147	0.9998	-	0.29 ± 0.01	0.01 ^i^	-	29
15	3-(Methyl-thio)-1-propanal	2-Methyl-3-heptanone	104	57	3.4317	0.9989	0.13 ± 0.04	0.04 ± 0	0.00043 ^g^	313	83
25	3-(Methyl-thio)-1-propanol	2-Methyl-3-heptanone	106	57	1.4308	0.9999	0.12 ± 0.02	0.01 ± 0	0.036 ^g^	3	<1
41	Lenthionine	2-Methyl-3-heptanone	142	57	0.2681	0.9998	1.09 ± 0.03	3.97 ± 0.04	0.00027 ^j^	4048	14,713
Alcohols											
10	1-Hexanol	2-Nonanol	69	97	0.2128	0.9909	0.03 ± 0	0.36 ± 0.08	0.5 ^e^	<1	<1
16	1-Octen-3-ol	2-Nonanol	57	97	0.0991	0.9999	157.65 ± 1.17	0.44 ± 0.08	0.0015 ^f^	105,100	294
17	2-Ethyl-1-hexanol	2-Nonanol	57	97	0.0811	0.9999	-	0.14 ± 0.03	25.4822 ^f^	-	<1
21	(E)-2-Octen-1-ol	2-Nonanol	57	97	0.1390	0.9983	0.05 ± 0.01	0.18 ± 0.05	0.84 ^k^	<1	<1
30	Phenylethyl alcohol	2-Nonanol	91	97	0.0416	0.9914	0.57 ± 0.01	9.20 ± 2.10	0.56423 ^f^	1	16
Acids											
14	Acetic acid	2-Ethylbutyric acid	60	88	1.2206	0.9971	-	177.54 ± 2.78	99 ^g^	-	2
24	Isovaleric acid	2-Ethylbutyric acid	60	88	0.4357	0.9958	0.24 ± 0.01	7.06 ± 0.28	0.49 ^g^	<1	14
34	Octanoic acid	2-Ethylbutyric acid	60	88	0.8162	0.9937	0.52 ± 0.03	-	3 ^e^	<1	-
37	Benzoic acid	2-Ethylbutyric acid	105	88	0.7957	0.9958	-	5.28 ± 0.21	1 ^l^	-	<1
Furanones											
33	4-Hydroxy-2,5-dimethyl-3(2H)-furanone	2-Ethylbutyric acid	128	88	5.1975	0.9984	-	1.43 ± 0.18	0.04 ^g^	-	36
35	3-Hydroxy-4,5-dimethyl-2(5H)-furanone	2-Ethylbutyric acid	128	88	6.9541	0.9991	0.02 ± 0	0.09 ± 0.01	0.00049 ^g^	39	187

^a^ Selected EI-MS ion for target compound used for response factor and quantitation. ^b^ Selected EI-MS ion of internal standard. ^c^ *R*^2^ values for linear regression of standard curves.^d^ Odor activity value (ratio of concentration to odor threshold). ^e^ Odor thresholds in water according to Pino and Mesa [40]. ^f^ Odor thresholds in water according to Giri et al. [41]. ^g^ Odor thresholds in water according to Czerny et al. [42]. ^h^ Odor thresholds in water according to Buttery [43]. ^i^ Odor thresholds in water according to Buttery et al. [44]. ^j^ Odor thresholds in water according to Schmidberger and Schieberle [3]. ^k^ Odor thresholds in water according to Eriksson et al. [45]. ^l^ Odor thresholds in water according to Escudero et al. [46].

In the dried shiitake mushrooms, there were 22 predominant odorants, of which ethyl acetate (218.34 ± 16.35 µg/g) had the highest concentration, followed by acetic acid (177.54 ± 2.78 µg/g), phenylethyl alcohol (9.20 ± 2.10 µg/g) and isovaleric acid (7.06 ± 0.28 µg/g). Phenylethyl alcohol and isovaleric acid were only reported to show higher contents in the dry *Flammulina velutipes* [47]. Lenthionine had the highest OAV of 14713 and was considered as the most important contributor, followed by 1-octen-3-one (1989) and phenylacetaldehyde (422). This illustrated that whatever the fresh or dried shiitake mushrooms, lenthionine and 1-octen-3-one contributed the main flavor to the overall aroma.

In addition, there was some inconsistency between DF determined by DFA, FD determined by AEDA and OAV. In the fresh shiitake mushrooms, benzaldehyde and (E)-2-octen-1-ol had the highest DF factors (DF = 8), and isovaleric acid and octanoic acid had relatively high FD factors (FD = 27), but they all had lower OAVs (OAVs < 1). Similarly, 2-ethyl-1-hexanol, benzaldehyde and (E)-2-octen-1-ol had the highest DF factors (DF = 8), and 1-hexanol and 3-(methyl-thio)-1-propanol had relatively high FD factors (FD = 27), but they all had lower OAVs (OAVs < 1) in the dried shiitake mushrooms. This might be due to DFA and AEDA were carried out on the extracts of shiitake mushrooms, which eliminated the influence of food matrix. However, OAV took into account the effect of the food matrix [31]. This inconsistency indicated that the food matrix affected the contribution of volatile components to the aroma. Moreover, ethyl acetate, acetic acid, phenylethyl alcohol and 3-hydroxy-4,5-dimethyl-2(5H)-furanone were identified as predominant odorants in dried shiitake mushrooms by AEDA and their OAVs were greater than or equal to 1, but DFA showed they were non-essential. 1-Octen-3-ol had relatively high OAV and DF factor, but it was not identified as predominant odorants by AEDA in the fresh samples. This might be due to the fact that DFA and AEDA were based on different extraction methods, HS-SPME and DSE-SAFE. HS-SPME specializes in extracting highly volatile compounds and DSE-SAFE extracted semi-volatile and volatile compounds [21]. Thus, the combination of DFA and AEDA could more comprehensively identify the odorants of shiitake mushrooms.

On the basis of OAV ≥ 1, 3-methyl-butanal (chocolate), octanal (citrus-like), (E)-2-octenal (fatty, nutty), phenylacetaldehyde (floral), *trans*-4,5-epoxy-(E)-2-decenal (metallic), 3-octanone (mushroom), 1-octen-3-one (mushroom), 3-(methyl-thio)-1-propanal (cooked potato-like), 3-(methyl-thio)-1-propanol (cabbage), lenthionine (sulfury, burnt), 1-octen-3-ol (mushroom), phenylethyl alcohol (honey) and 3-hydroxy-4,5-dimethyl-2(5H)-furanone (seasoning-like) were considered to be the key contributors to the aroma of fresh shiitake mushrooms, and ethyl acetate (pineapple), 3-methyl-butanal (chocolate), phenylacetaldehyde (floral), 2,3-butanedione (sweet, creamy), 1-octen-3-one (mushroom), dimethyl trisulfide (sulfury), 3-(methyl-thio)-1-propanal (cooked potato-like), lenthionine (sulfury, burnt), 1-octen-3-ol (mushroom), acetic acid (vinegar-like), isovaleric acid (sweaty), phenylethyl alcohol (honey), 3-hydroxy-4,5-dimethyl-2(5H)-furanone (seasoning-like) and 4-hydroxy-2,5-dimethyl-3(2H)-furanone (caramel) were detected as the key contributing odorants in the dried shiitake mushrooms. 

### 3.4. Aroma Recombination Verification

**Figure 1:**
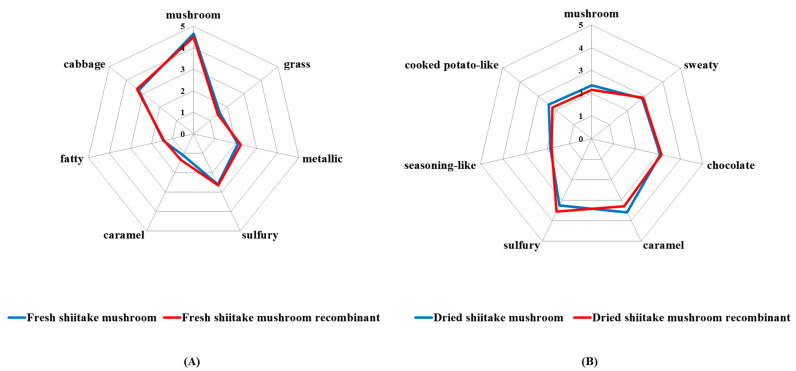
Aroma reconstitution models of fresh (**A**) and dried (**B**) shiitake mushrooms.

It was well accepted that the predominant odorants obtained by the OAV concept could be confirmed by aroma reconstitution experiments [48]. For this purpose, reconstitution models were prepared containing odorants with high OAVs (OAV ≥ 1) in their actual concentrations in the fresh and dried shiitake mushrooms, respectively. The similarities between the recombination and original samples were judged by the included angle cosine analysis [20]. The results for aroma are summarized in Figure 1, and show that the average similarity degrees were 0.9987 for the fresh samples, and 0.9977 for the dried samples. Thus, it was corroborated that the identification and quantitation of predominant odorants of the fresh and dried shiitake mushrooms could be considered successful, due to the fact that the reconstitution odorants exhibited high similarities to the original samples.

Besides, it is shown in (Figure 1) that the mushroom-like odor was reduced after drying. This was due to the decreased OAVs of 3-octanone, 1-octen-3-one and 1-octen-3-ol after drying, which contribute mushroom-like odor. Moreover, the sulfury odor was more intense after drying. This was related to the fact that dimethyl trisulfide and lenthionine had higher OAVs in dried shiitake mushrooms. Similarly, the enhancement of sweaty and caramel-like odors after drying might be due to the increased OAVs of isovaleric acid and 4-hydroxy-2,5-dimethyl-3(2H)-furanone, respectively. 

### 3.5. Variations and Aroma Chemistry of the Key Contributing Odorants in Shiitake Mushrooms after Hot-Air-Drying

**Figure 2:**
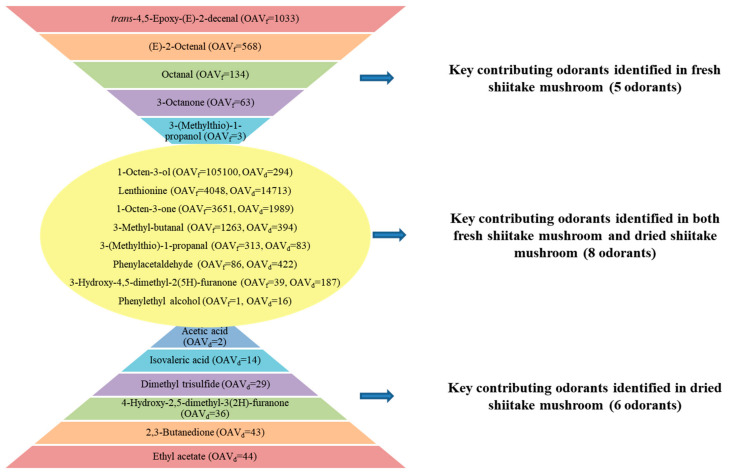
Distribution of key contributing odorants (OAV ≥ 1) identified in the fresh and dried shiitake mushrooms. OAV, odor activity value; OAV_f_, OAV in the fresh shiitake mushrooms; OAV_d_, OAV in the dried shiitake mushrooms.

**Figure 3:**
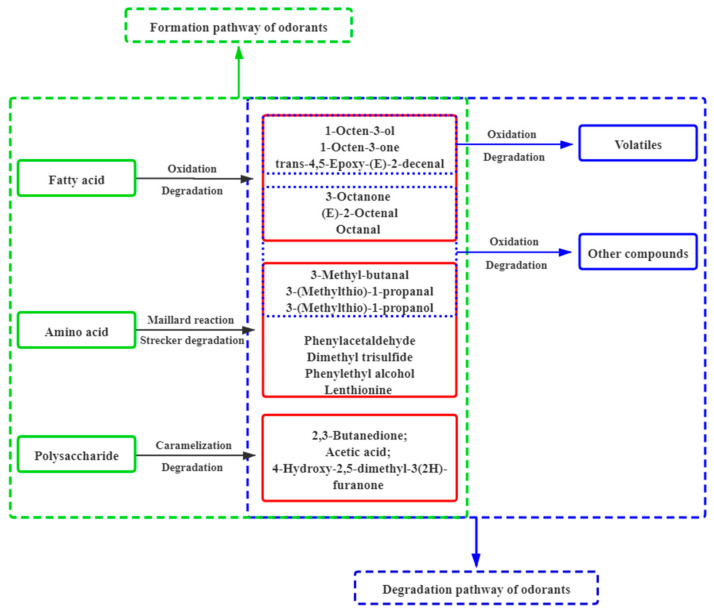
Aroma chemistry involved in the formation and degradation of key contributing odorants in shiitake mushrooms.

Figure 2 shows the key contributing odorants in shiitake mushrooms before and after dying and (Figure 3) illustrates the aroma chemistry of the changes.

Some key contributing odorants in fresh shiitake mushrooms were not detected after drying, including *trans*-4,5-epoxy-(E)-2-decenal (metallic), (E)-2-octenal (fatty, green), octanal (citrus-like), 3-octanone (mushroom) and 3-(methyl-thio)-1-propanol (cooked potato-like). This further illustrated the reasons for the decrease in mushroom-like, metallic, green and fatty characteristics of the aroma after drying (Figure 1A). The loss of odorants might be due to thermal decomposition and oxidation during the drying process, which generated volatile or other compounds [12,49]. 

Moreover, there were an additional six key contributing odorants detected in shiitake mushrooms after drying, including ethyl acetate (pineapple), 2,3-butanedione (sweet, creamy), dimethyl trisulfide (sulfury), acetic acid (vinegar-like), isovaleric acid (sweaty) and 4-hydroxy-2,5-dimethyl-3(2H)-furanone (caramel). This is the reason for the increase in sulfury, caramel-like and sweaty characteristics of the aroma after drying (Figure 1B). These compounds might be generated from fatty acids by oxidation or degradation, amino acids by Maillard reaction or Strechker and polysaccharides by caramelization or degradation (Figure 3). For example, 4-hydroxy-2,5-dimethyl-3(2H)-furanone could be formed via carbohydrate degradation reactions [50] and 2,3-butanedione and acetic acid were formed via caramelization reactions [21,51]. 

**Figure 4:**
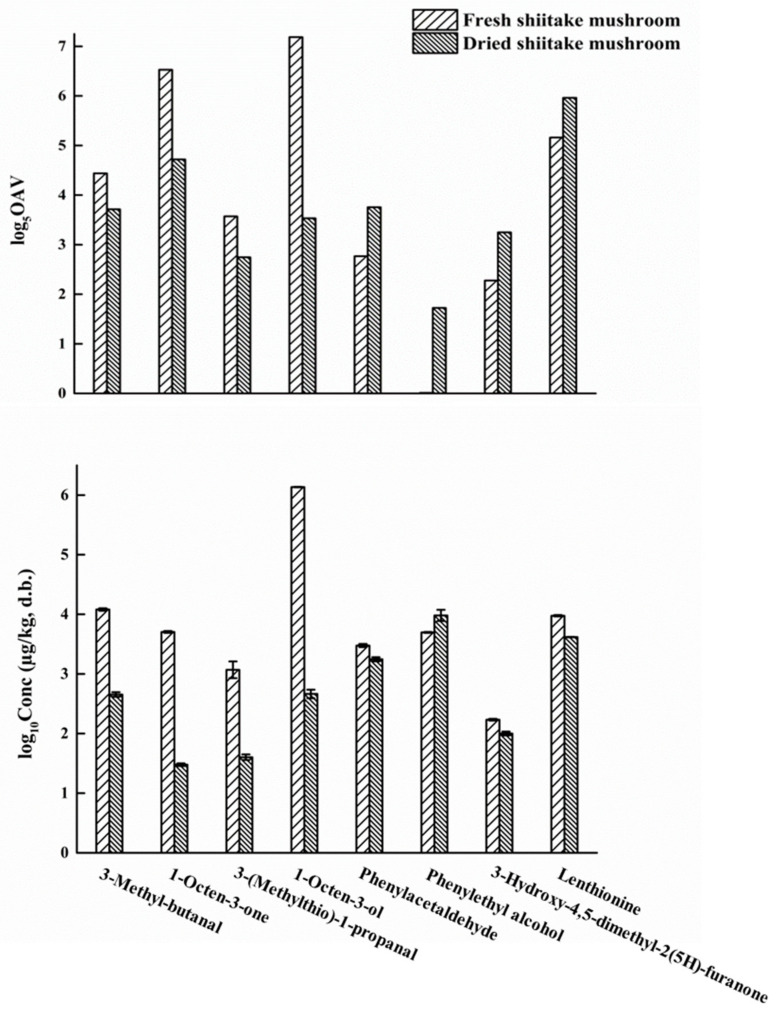
Odor activity values (log_5_OAV) and concentrations on a dry basis (log_10_ Conc (µg/kg, d.b.)) of the key contributing odorants (OAV ≥ 1) in both fresh and dried shiitake mushrooms.

**Figure 5:**
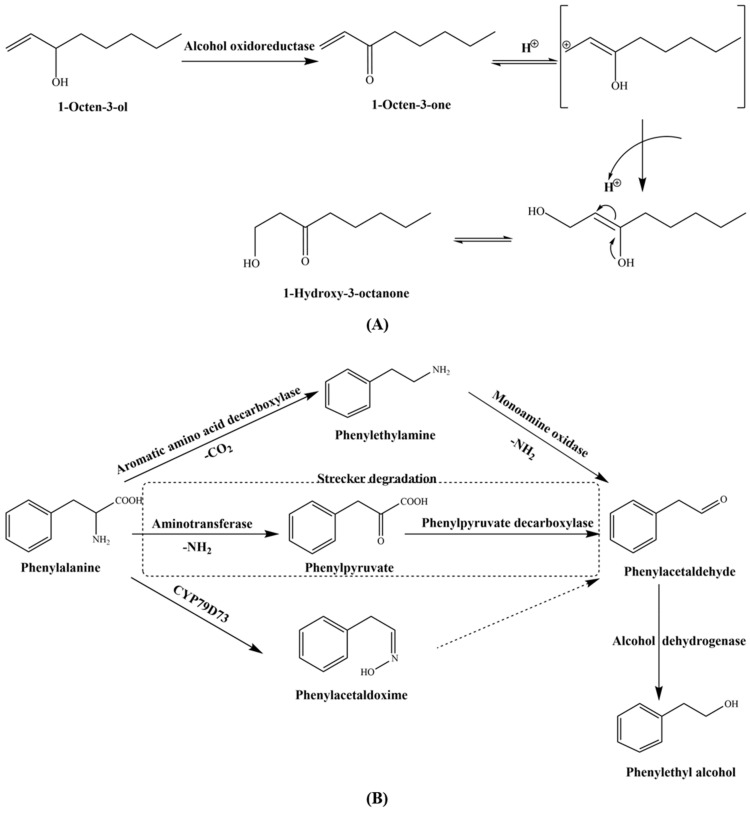
Main pathways involved in the degradation of 1-octen-3-ol (**A**) and the formation of phenylethyl alcohol (**B**) in shiitake mushrooms during drying.

There were also eight key contributing odorants detected both before and after drying, including 1-octen-3-ol, lenthionine, 1-octen-3-one, 3-methyl-butanal, 3-(methyl-thio)-1-propanal, phenylacetaldehyde, 3-hydroxy-4,5-dimethyl-2(5H)-furanone and phenylethyl alcohol. However, their concentrations and OAVs also changed after drying as shown in (Figure 4). Among these eight odorants, 1-octen-3-ol (mushroom) showed the most significant decrease in concentration, and also in OAV, which was reduced by 357 times. The degradation of 1-octen-3-ol in shiitake mushrooms during drying is displayed in (Figure 5A). Firstly, 1-octene-3-ol generated 1-octene-3-one under the action of alcohol oxidoreductase. Subsequently, 1-octene-3-one might be prone to the aldol-type reactions to form 1-hydroxy-3-octanone during thermal treatment of foods [3,52]. Moreover, the OAV of phenylethyl alcohol (honey) increased the most, by 16 times, after drying. There were three probable formation pathways of phenylethyl alcohol in shiitake mushrooms during drying (Figure 5B). As the precursor of phenylethyl alcohol, phenylalanine first generated phenylacetaldehyde, as an intermediate, through three pathways, with the participation of various enzymes required [53,54]. The first pathway was that phenylalanine was decarboxylated by aromatic amino acid decarboxylase to produce phenylethylamine, which was then deaminated by monoamine oxidase to generate phenylacetaldehyde [53]. The second pathway was the Strecker degradation of phenylalanine. This was deaminated by aminotransferase to generate phenylpyruvate, and then formed phenylacetaldehyde via phenyl-pyvate decarboxylase [53]. The third pathway was phenylalanine produced phenlacetaldoxime by CYP97D73, and then produced phenylacetaldehyde [54]. Subsequently, phenylacetaldehyde was converted into phenylethyl alcohol with alcohol dehydrogenase [53]. Besides, the OAVs of lenthionine (sulfury) and 3-hydroxy-4,5-dimethyl-2(5H)-furanone (seasoning-like) increased. This also improved the sulfury and seasoning characteristics of the aroma after drying.

## 4. Conclusions

A comprehensive molecular sensory science approach, including SPME, DSE-SAFE, GC-O, DFA, AEDA, OAV and aroma recombination verification, was explored to identify and quantify the predominant odorants and their contributions to the aroma of fresh and hot-air-dried shiitake mushrooms and their variations induced by hot-air-drying. 1-Octen-3-ol, lenthionine, 1-octen-3-one and *trans*-4,5-epoxy-(E)-2-decenal were the key contributing odorants in fresh shiitake mushrooms. This accounted for the fact that the shiitake mushrooms were characterized by mushroom-like, sulfury and metallic odor before hot-air-drying. After hot-air-drying, mushroom-like odor was diminished, following the decrease of 1-octen-3-ol, 1-octen-3-one and 3-octanone due to thermal decomposition and oxidation. However, sulfury, caramel, and seasoning-like odor stood out in the aroma due to the increase or formation of lenthionine, dimethyl trisulfide, 4-hydroxy-2,5-dimethyl-3(2H)-furanone and 3-hydroxy-4,5-dimethyl-2(5H)-furanone by the reactions of fatty acids, amino acids and polysaccharides. Ultimately, lenthionine became the most important contributor to aroma with the highest OAV, and the hot-air-dried shiitake mushrooms featured a sulfury odor. Overall, the predominant odorants contributing to the aroma of shiitake mushrooms before and after hot-air-drying were revealed at the molecular level. The contributions and variations in the odorants of shiitake mushrooms and their mechanism were preliminarily studied.

## Figures and Tables

**Table 1:** The concentrations of the reference odorants in different odor intensities.

Odor Descriptors	Reference Odorants	Concentrations in Water (µg/mL)					
		0 ^a^	1 ^a^	2 ^a^	3 ^a^	4 ^a^	5 ^a^
mushroom	1-Octen-3-ol	0	1.50 × 10^−2^	1.50 × 10^−1^	1.50	1.50 × 10	1.50 × 10^2^
grass	(E)-2-Octen-1-ol	0	33.60 × 2^−2^	33.60 × 2^−1^	33.60	33.60 × 2	33.60 × 2^2^
metallic	*trans*-4,5-Epoxy-(E)-2-decenal	0	0.13 × 10^−2^	0.13 × 10^−1^	0.13	0.13 × 10	0.13 × 10^2^
sulfury	Lenthionine	0	0.27 × 10^−2^	0.27 × 10^−1^	0.27	0.27 × 10	0.27 × 10^2^
caramel	4-Hydroxy-2,5-dimethyl-3(2H)-furanone	0	10.00 × 5^−2^	10.00 × 5^−1^	10.00	10.00 × 5	10.00 × 5^2^
fatty	(E)-2-Octenal	0	3.00 × 10^−2^	3.00 × 10^−1^	3.00	3.00 × 10	3.00 × 10^2^
cabbage	3-(Methyl-thio)-1-propanol	0	9.00 × 5^−2^	9.00 × 5^−1^	9.00	9.00 × 5	9.00 × 5^2^
chocolate	3-Methyl-butanal	0	1.10 × 10^−2^	1.10 × 10^−1^	1.10	1.10 × 10	1.10 × 10^2^
sweaty	Isovaleric acid	0	44.10 × 3^−2^	44.10 × 3^−1^	44.10	44.10 × 3	44.10 × 3^2^
seasoning-like	3-Hydroxy-4,5-dimethyl-2(5H)-furanone	0	0.49 × 10^−2^	0.49 × 10^−1^	0.49	0.49 × 10	0.49 × 10^2^
cooked potato-like	3-(Methyl-thio)-1-propanal	0	0.43 × 10^−2^	0.43 × 10^−1^	0.43	0.43 × 10	0.43 × 10^2^

^a^ The odor intensities from 0 to 5.

**Table 2:** Odorants determined in fresh and hot-air-dried shiitake mushrooms by HS-SPME and DSE-SAFE combined with DFA and AEDA.

No.	Compound	CAS	Odor ^a^	ID ^b^	RI ^c^		DF ^d^		FD ^e^	
					Wax	HP-5	Fresh	Dried	Fresh	Dried
1	2-Methyl-propanal	78-84-2	chocolate	RI,O,S	743	<700	-	-	27	3
2	Ethyl acetate	141-78-6	pineapple	RI,MS,O,S	887	-	-	-	-	27
3	3-Methyl-butanal	590-86-3	chocolate	RI,MS,O,S	919	<700	8	8	243	243
4	2,3-Butanedione	431-03-8	sweet, creamy	RI,MS,O	980	-	-	2	-	3
5	Dimethyl disulfide	624-92-0	cabbage	RI,MS,O,S	1069	747	-	-	-	9
6	3-Octanone	106-68-3	mushroom	RI,MS,O,S	1251	988	8	4	3	3
7	Octanal	124-13-0	citrus-like	RI,MS,O,S	1286	1006	6	-	3	-
8	1-Octen-3-one	4312-99-6	mushroom	RI,MS,O,S	1289	965	8	8	81	3
9	2-Acetyl-1-pyrroline	85213-22-5	popcorn-like	RI,O	1320	904	8	8	1	1
10	1-Hexanol	111-27-3	sweet, oily	RI,MS,O,S	1351	-	-	-	3	27
11	Dimethyl trisulfide	3658-80-8	sulfury	RI,MS,O,S	1365	962	-	8	-	81
12	(E)-2-Octenal	2548-87-0	fatty, nutty	RI,MS,O,S	1410	1064	-	-	9	-
13	Unknown	-	grass, beany	O	1428	-	8	8	243	-
14	Acetic acid	64-19-7	vinegar-like	RI,MS,O,S	1432	<700	-	-	1	81
15	3-(Methyl-thio)-1-propanal	3268-49-3	cooked potato-like	RI,MS,O,S	1447	-	8	8	243	3
16	1-Octen-3-ol	3391-86-4	mushroom	RI,MS,O,S	1456	977	8	8	81	1
17	2-Ethyl-1-hexanol	104-76-7	fresh, fatty	RI,MS,O,S	1482	1031	2	8	-	9
18	Benzaldehyde	100-52-7	almond, green	RI,MS,O,S	1512	920	8	8	3	1
19	(E)-2-Nonenal	18829-56-6	fatty, green	RI,MS,O,S	1524	1156	-	6	1	1
20	1-Octanol	111-87-5	green, fresh	RI,MS,O,S	1545	1070	-	-	1	-
21	(E)-2-Octen-1-ol	18409-17-1	grass	RI,MS,O,S	1614	-	8	8	-	-
22	Butanoic acid	107-92-6	cheese, fecal	RI,MS,O,S	1620	797	6	6	-	1
23	Phenylacetaldehyde	122-78-1	floral	RI,MS,O,S	1633	1041	8	8	729	81
24	Isovaleric acid	503-74-2	sweaty	RI,MS,O,S	1673	843	-	8	27	81
25	3-(Methyl-thio)-1-propanol	505-10-2	cabbage	RI,MS,O,S	1709	-	8	6	3	27
26	1,2,4-Trithiolane	289-16-7	mushroom	RI,MS,O	1721	1093	4	4	3	3
27	Pentanoic acid	109-52-4	sweaty, rancid	RI,MS,O,S	1744	-	4	-	-	-
28	Dimethyl tetra-sulfide	5756-24-1	mushroom	RI,MS,O	-	1205	4	6	243	729
29	Unknown	-	rubber	O	1787	-	3	-	-	-
30	Phenylethyl alcohol	60-12-8	honey	RI,MS,O,S	1897	1119	-	6	81	27
31	Unknown	699-10-5	mushroom	MS,O	1953	-	3	-	1	3
32	*trans*-4,5-Epoxy-(E)-2-decenal	134454-31-2	metallic	RI,MS,O,S	1994	1383	8	-	729	-
33	4-Hydroxy-2,5-dimethyl-3(2H)-furanone	3658-77-3	caramel	RI,MS,O,S	2024	1063	-	8	1	729
34	Octanoic acid	124-07-2	sweaty, fatty	RI,MS,O,S	2087	1176	6	-	27	1
35	3-Hydroxy-4,5-dimethyl-2(5H)-furanone	28664-35-9	seasoning-like	RI,O,S	2191	1084	6	6	81	81
36	1,2,4,5-Tetrathiane	291-22-5	sulfury, burnt	RI,MS,O	2207	1335	8	8	729	729
37	Benzoic acid	65-85-0	balsam-like	RI,MS,O,S	2400	-	-	-	-	9
38	1, 2, 4, 6-Tetrathiepane	292-45-5	sulfury	RI,MS,O	2445	1498	6	2	729	27
39	Phenylacetic acid	103-82-2	floral	RI,MS,O	2561	1241	4	8	81	27
40	Unknown	-	fecal	O	2574	-	-	-	27	27
41	Lenthionine	292-46-6	sulfury, burnt	RI,MS,O	2638	1619	8	8	729	729

HS-SPME, headspace solid phase microextraction; DSE-SAFE, direct solvent extraction combined with solvent-assisted flavor evaporation; DFA, detection frequency analysis; AEDA, aroma extract dilution analysis. ^a^ Odor description perceived at the sniffing port. ^b^ Identification methods: retention index (RI), odor quality (O), mass spectra (MS) and standards (S). ^c^ Retention index. ^d^ Detection frequency factors. ^e^ Flavor dilution factors.

## Data Availability

The data showed in this study are contained within the article.
